# Development of a survey tool to measure pediatric experience of care: Cognitive testing and validation in the Laos

**DOI:** 10.1371/journal.pgph.0006108

**Published:** 2026-06-30

**Authors:** Teemar Fisseha, Elizabeth Bunde, Phoutthasone Phimmakaisone, Emma Sacks, Nancy Vollmer, Kate Gilroy, Bouangern Xayalath, Bandith Soumphonphakdy, Soumya Alva

**Affiliations:** 1 International Division, JSI Research and Training Institute, Inc., Washington, District of Columbia, United States of America; 2 International Division, JSI Research and Training Institute, Inc., Vientiane, Lao People’s Democratic Republic; 3 Department of International Health, Johns Hopkins School of Public Health, Baltimore, Maryland, United States of America; 4 Maternal Child Health Center, Ministry of Health, Vientiane, Lao People’s Democratic Republic; University of Sydney, AUSTRALIA

## Abstract

Despite increased availability of child health services, poor quality of care remains a major barrier to improved health outcomes in low-resource settings. Few validated tools exist to measure pediatric experience of care (PEC). Building on prior work in respectful maternity care, this study developed a caregiver-reported PEC survey based on a new conceptual framework and assessed the clarity, relevance, and cultural appropriateness of survey items through cognitive testing in Laos. Qualitative cognitive testing was conducted in May 2023 with 34 caregivers of children under five in Vientiane and Oudomxay provinces. Five rounds of interviews were completed. Lao-speaking researchers used structured probes and open-ended questions to assess comprehension, cultural fit, and interpretation of items. Daily debriefings informed iterative revisions. Interview notes were analyzed using three types of potential response error: unclear or ambiguous question wording; translation and linguistic challenges; and cultural relevance. Respondents had difficulty interpreting abstract or subjective concepts, such as “respect,” “trust,” or perceptions of privacy or wait time. Items using such constructs were revised to reference concrete, observable experiences (e.g., quantifying wait times; describing “kindness” instead of “respect”). Translation challenges also shaped understanding; respondents preferred common terms such as “health worker” and cognitively oriented language (“think”) rather than emotional wording (“feel”). Several concepts, such as “building trust,” had limited cultural resonance; revised items emphasized behavioral indicators (e.g., whether the child was helped to feel calm). These adaptations improved clarity, cultural alignment, and response validity. Cognitive testing demonstrated that valid measurement of PEC requires more than accurate translation; it demands cultural and linguistic adaptation reflecting local norms and communication practices. The study confirms the feasibility and necessity of contextually grounded PEC measurement in low-resource settings and reinforces the importance of iterative, culturally responsive instrument development to ensure meaningful, actionable data for improving child health services.

## Background

Reducing preventable child deaths remains a global priority, yet disparities in survival persist, particularly in low- and lower-middle-income countries (LMICs), with sub-Saharan Africa and Southern Asia notably lagging in reducing under-five mortality [1,2]. Expanding access to child health services alone is insufficient to ensure improved outcomes; the quality of care and the experience of patients within health systems are increasingly recognized as critical factors [3–7].

Poor quality of care contributes not only to sub-optimal health outcomes but also erodes trust in the health systems, deters future care-seeking, and exacerbates existing health inequities, and can limit service coverage [8–11]. Evidence shows that quality is a critical driver of health outcomes, and that investments in access will fall short unless accompanied by efforts to improve how care is delivered and experienced [12]. Despite widespread improvements in service readiness and availability of child health services in LMICs, poor quality of care continues to limit effective coverage, highlighting that these factors alone are insufficient to improve child health outcomes [13,14].

As part of this shift, global and national health strategies are increasingly emphasizing “experience of care” as a critical dimension of quality [6,7]. This includes respectful, dignified interactions with health providers; clear communication; emotional support; and the availability and cleanliness of the health environment [15]. These interpersonal and facility-level factors directly influence whether individuals choose to seek care, adhere to treatment, or return for future services. While efforts to measure experience of care have expanded, particularly in maternal health with a focus on women’s experiences during labor and delivery, there remains a major gap in validated tools and standardized approaches for measuring experience of care in pediatric populations [5,16].

This gap is particularly acute in LMICs, where tools to assess pediatric experience of care are often lacking, underdeveloped, or not adapted to the realities of diverse health system settings. Most available tools were developed in high-income contexts and are frequently applied without adequate cultural or contextual adaptation, limiting their validity and usefulness in LMICs [15,17]. Without reliable ways to measure and understand children’s and caregivers’ experiences in the health system, countries are limited in their ability to identify service gaps, improve quality, and track progress towards health goals [18,19].

**Fig 1:**
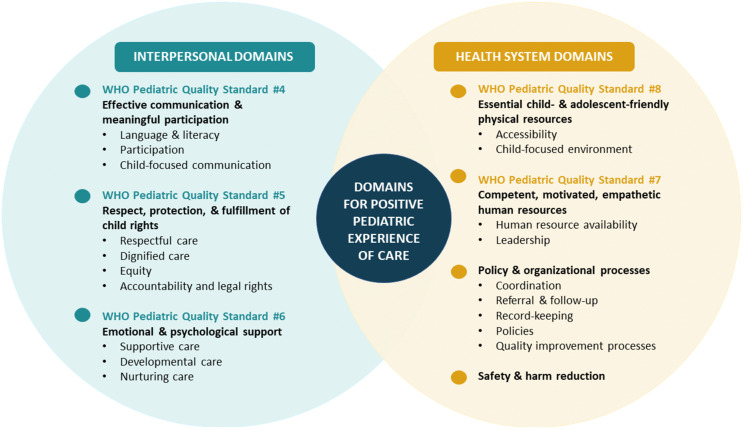
Pediatric experience of care framework.

To address these gaps, the MOMENTUM Knowledge Accelerator (MKA) project conducted a scoping review of existing frameworks and tools and developed a pediatric experience of care (PEC) framework, grounded in WHO quality standards [20]. The PEC framework encompasses three interpersonal domains (effective communication, respect for child rights, and emotional support), as well as four system-level domains (child-friendly resources, competent and empathetic staff, supportive policies, and safety and harm reduction), and provides a conceptual foundation for developing context-specific measures (Fig 1). Building on this framework, MKA developed a pediatric proxy-report survey designed for caregivers to evaluate the care experiences of children under five. The tool incorporates validated items from existing instruments and structured to assess care experiences across service types, facility levels, and visit contexts [21–25].

The purpose of this study was to conduct qualitative cognitive testing interviews with caregivers of children under five to assess the clarity, relevance, and interpretability of items in a draft PEC survey. Cognitive testing aimed to determine whether caregivers understood the questions and response options as intended, to identify sources of confusion or misinterpretation, and to assess whether survey items accurately captured the intended concepts across PEC domains.

Laos was selected for cognitive testing due to the intersection of persistent child health challenges, substantial sociolinguistic diversity, and ongoing health system reforms under universal health coverage (UHC). Despite major investments in expanding primary health care and implementing the National Health Insurance scheme, under-five mortality in Laos remains among the highest in Southeast Asia, driven largely by preventable conditions including neonatal complications, pneumonia, and diarrheal disease [26,27]. These disparities are particularly pronounced among rural, low-income, and ethnic minority populations, who continue to experience barriers related to geography, language, and health system access [28].

At the same time, Laos provides a relevant setting for evaluating pediatric PEC measures because the public health sector delivers the majority of child health services nationwide, making caregiver perceptions especially important for understanding utilization and continuity of care. Previous research in LMICs has shown that expanded coverage alone does not necessarily improve outcomes when quality and patient experience remain weak [29–31].

## Methods

We conducted cognitive testing with caregivers of children under age five (n = 34) to evaluate the clarity, relevance, and cultural appropriateness of survey items in a draft PEC survey. Cognitive testing was implemented through individual interviews, using probing questions to identify points of confusion or misinterpretation and ensure items captured the intended concepts across the PEC framework’s domains.

### Study timeline

The study was conducted between 24/05/2023 and 05/06/2023. Training of the research team took place from 24/05/2023–26/05/2023 in Vientiane capital and included a pilot test in Vientiane province. The pilot was conducted using a convenience sample of individuals present at the selected facilities during the visit; therefore, no formal recruitment period applied for the pilot phase.

The main recruitment and data collection activities were conducted in Oudomxay province from 29/05/2023–02/06/2023. Prior to the research team’s arrival, facility staff were notified and reviewed facility registers to identify eligible caretakers according to the predefined selection criteria. On the day of each facility visit, the research team performed random selection of participants from the list of eligible caretakers. Accordingly, the recruitment period for the main study commenced on 29/05/2023 and concluded on 02/06/2023. A final study debriefing was conducted on 05/06/2023 upon return from the field.

### Study setting

The study included a pilot phase in Vientiane Province (excluding the capital) to refine the survey tool, followed by the main study in Xay and Beng districts of Oudomxay Province to assess broader feasibility and applicability. Results from both the pilot and main sites were analyzed to inform iterative tool refinement and assess overall clarity, relevance, and cultural appropriateness.

Vientiane was selected due to its proximity to the training site and its representation of areas with better access to health services, particularly in the capital region. With a largely rural population of about 419,000, Vientiane Province performs better than the national average in vaccination and early childhood development but faces higher mortality and nutritional challenges [32,33]. Xay and Beng districts in Oudomxay were chosen to represent rural and peri-urban areas to provide a contrast in health care access and outcomes. Oudomxay, one of the poorest provinces in Laos with a population of nearly 308,000, experiences worse child health outcomes, including higher mortality rates and nutrition-related issues than the national average, particularly in its rural districts [34]. Xay is more urbanized, while Beng remains predominantly rural, with a strong reliance on subsistence farming [17].

### Tool development

We developed a survey tool aligned with the PEC framework to capture the seven core domains essential for a positive care experience: (1) effective communication; (2) respect for child rights; (3) emotional support; (4) child-friendly resources; (5) competent and empathetic staff; (6) supportive policies; and (7) safety and harm reduction.

The initial draft survey comprised 77 closed-ended and four open-ended questions designed to capture caregivers’ experiences. Of these, 57 questions applied to both inpatient and outpatient care, while 20 were specific to inpatient services. As none of the participating facilities provided inpatient care, only the 57 applicable items underwent cognitive testing (S1 File). Following iterative rounds of cognitive testing, the final instrument included 64 closed-ended items, using binary (yes/no) and four-point Likert scale response options, alongside six open-ended questions to elicit richer narrative data. The increase in the total number of items reflects the disaggregation of multi-part questions into separate items for clarity, as well as the addition of new questions (S2 File). The survey was initially developed in English, translated into Lao by a professional translator, and further refined through an iterative process that began during enumerator training and continued through pilot testing and field interviews.

### Research team

Cognitive testing interviews were conducted by four experienced, native Lao-speaking qualitative researchers, organized into two teams, each with a lead facilitator and a note-taker. A three-person supervision team oversaw the process, leading the training, daily debriefings, and guiding tool revisions. All members of the field team were bilingual in Lao and English, enabling them to suggest alternative phrasing and nuanced interpretations for the research team to consider during tool refinement. The local research team’s cultural knowledge was integral to the analytic process. During daily debriefings, local team members actively contributed cultural interpretation and contextual insight, enabling the team to distinguish between linguistic and cultural sources of response error. This ensured that cultural influences on item interpretation were identified by culturally embedded practitioners drawing on lived experience and direct field observation, rather than inferred solely from existing literature on Lao society. The team underwent a three-day training on interviewing principles, tool review, informed consent procedures, and effective interviewing techniques. A pilot test was also conducted with caregivers in Vientiane province as part of the training.

### Study design and sample

The study employed cognitive testing to evaluate the draft PEC survey tool. This qualitative method is commonly used in instrument development to assess whether survey items are understood as intended by the target population [35]. It focuses on how respondents comprehend, interpret, and respond to questions and response options, identifying potential sources of confusion, cultural misalignment, or misinterpretation. This approach was chosen to ensure the tool’s content validity and contextual appropriateness before broader implementation in real-world health settings.

Public health facilities in both provinces were purposively selected, in collaboration with their respective Provincial Health Offices (PHO), based on geographic accessibility, service volume, and availability of pediatric care to ensure appropriate participants for the cognitive testing study. Testing in public health facilities was particularly valuable, as most child health services in Laos are delivered through the public sector. This approach ensured the tool reflected real-world service delivery, enhanced policy relevance and feasibility for national implementation, and fostered local ownership among providers and caregivers. Private health facilities in Laos are limited in number and concentrated primarily in urban areas, particularly Vientiane capital, making public facilities the most relevant and representative setting for a tool intended for broad national implementation. Insights from this study are also relevant for other contexts where public facilities dominate service delivery, offering lessons for adapting and institutionalizing patient experience measures in government health systems.

Selection criteria included adults aged 18 or older, male or female, who had sought sick or well-child care for a child under five in the past six months and lived within the facility’s catchment area. Caregivers did not need to be the child’s parent. The study focused on children under five attending outpatient services because this age group bears the highest burden of preventable morbidity and mortality and relies entirely on caregivers for health care decisions, making caregiver-reported surveys appropriate. Outpatient visits are the most common point of contact with the health system, providing a relevant setting to assess routine pediatric care quality. This focus also aligns with global health initiatives and allows for a manageable, methodologically sound approach to cognitive testing before adapting the tool for older children or inpatient care.

At the facility sites in Vientiane Province, caregivers were selected for the initial pilot phase using a convenience sample from those visiting that day for well- or sick-child services, while the research team was present for pilot testing. The sample size was not pre-set and depended solely on caregiver attendance during the visit, resulting in a total of seven respondents. In Oudomxay Province, facility staff reviewed client registers to compile a list of eligible caregivers based on the selection criteria. The research team then randomly selected a total of 27 caregivers, consistent with other cognitive interview studies [36]. Facility staff helped contact clients and assess interest in participation, with replacements randomly selected from the same list. Each interview in each round involved a different caregiver. The total sample size from Vientiane and Oudomxay was 34 caregivers (Table 1).

**Table 1:** Sample distribution.

Location	Urban	Rural	Total
**Vientiane Province (pilot)**			
Xaythany District Hospital	4	–	4
Khoksivilay Health Center	1	2	3
**Sub-Total**	**5**	**2**	**7**
**Oudomxay Province**			
Xay district: Phon Hom Health Center	6	8	14
Beng district: Na Ngua Health Center	3	10	13
**Sub-Total**	**9**	**18**	**27**
**Total**	**14**	**20**	**34**

While saturation was not formally defined a priori, the iterative structure of the five testing rounds served a functionally similar purpose. By approximately round three, patterns of comprehension difficulty were becoming consistent across participants, with fewer new issues emerging in each subsequent round. By round four, the team determined that major sources of confusion had been identified and addressed, and the fifth round confirmed stability in responses. Given the geographic dispersion of field sites in Oudomxay Province, research teams worked across locations simultaneously rather than sequentially, and saturation was therefore assessed retrospectively across rounds through the daily debriefing process rather than used prospectively to guide cessation of recruitment.

### Data collection

Interviews were conducted in a private area of the respondent’s home after obtaining verbal consent and permission to audio-record, which was documented on the tool and witnessed by a research team member. Only one participant declined recording but agreed to proceed with the verbal interview. The facilitator led the interviews and follow-up questions, while the notetaker managed recordings and documented responses, observations, and explanations. Both team members independently recorded responses for accuracy.

During each interview, the facilitator read each question aloud and used structured cognitive probes to assess comprehension and response validity (Table 2). Open-ended questions were added as needed to explore caregiver understanding and experience. Cognitive testing was conducted over five iterative rounds, with approximately seven caregivers interviewed per round (one round included six respondents). In each round, participants were asked the same structured set of cognitive probes focused on item relevance, clarity of question wording, and clarity of response options. Findings from each round informed iterative revisions to the survey, which were then re-tested in subsequent rounds using the same probes.

**Table 2:** Cognitive testing probes used to assess survey items.

Probe focus	Example question	Purpose
**Relevance**	“Is this item relevant or important to you?” (Response: yes/ no/ somewhat)	To assess whether the item reflects caregivers’ real experiences or priorities
**Clarity of wording**	“Is the question clear and understandable the way it is written?” (Response: yes/ no; if no, how would you change it?)	To identify unclear or confusing phrasing
**Clarity of response options**	“Are the response options clear?” (Response: yes/ no; if no, how would you improve them)	To check if caregivers could easily select an appropriate response

### Data management and analysis

At the end of each day, the full research team, including the Lao national data collectors, met to review responses, assess question and response option performance, and determine whether revisions were needed. These daily debriefings allowed the team to reflect on question clarity, respondent understanding, and item relevance, incorporating both participant responses and cultural and linguistic knowledge of the Lao national team. Based on these discussions, edits were made to the instrument, and the revised version was used in the next round of interviews. Note-takers transcribed interview notes while listening to the recordings to ensure accurate capture of details, nuances, and contextual highlights, helping to validate the data and provide a fuller account of each interview.

To guide analysis, we drew from established typology frameworks commonly used in similar studies, including the Cross-National Error Source Typology, to identify and classify sources of measurement error [37,38]. These frameworks offered a systematic, theory-informed approach to understanding how respondents interpreted and answered questions, allowing us to detect not only problematic items but the underlying causes of confusion or misinterpretation. Issues were categorized into three domains: (1) question clarity, including unclear wording or vague phrasing; (2) translation and linguistic accuracy, capturing distortions caused by incorrect or overly literal translations; and (3) cultural appropriateness and relevance, reflecting misunderstandings due to unfamiliar or context-inappropriate terms or concepts.

While many issues aligned clearly with a single domain, others spanned multiple areas. In these cases, we prioritized the primary cause while noting overlaps, ensuring both consistency and nuance in refining the tool. All data, including interview notes, tool revisions, and audio files, were securely stored on a password-protected cloud-based platform, accessible only to the core research team, ensuring participant confidentiality and data integrity.

### Ethical considerations

The study was reviewed by the Lao National Ethics Committee for Health Research and determined exempt from full ethical review (Approval #19, 2022). Additional authorization was obtained from the Ministry of Health’s Department of Health and Hygiene Promotion, the Department of Healthcare and Rehabilitation, and the Vientiane and Oudomxay Provincial and District Health Offices. All research team members were trained in ethical practices and informed consent, following international standards set by the 1964 Declaration of Helsinki and its later amendments. There were no deviations from the approved study protocol.

Participants were caregivers of children under five years of age recruited through health facilities in Vientiane and Oudomxay provinces. No minors were recruited or interviewed. Verbal informed consent, including permission to audio-record, was obtained from each participant in the Lao language before participation began. The consent process, covering the study’s purpose, procedures, risks, and voluntary nature, was read aloud to respondents, with consent documented in the survey tool and the interviewer serving as witness.

Verbal informed consent, including permission to audio-record, was obtained from each participant in Lao before interviews began. The consent process, covering the study’s purpose, procedures, risks, and voluntary nature, was read to respondents with consent documented in the survey tool and the interviewer serving as witness. No minors were recruited or interviewed. All research team members were trained in ethical practices and informed consent, following international standards set by the 1964 Helsinki Declaration and its later amendments.

This study was supported by two USAID-funded projects. The first is the MKA, part of the MOMENTUM suite of awards, implemented by Population Reference Bureau (PRB) with partners JSI Research and Training Institute, Inc. and Ariadne Labs under Cooperative Agreement #7200AA20CA00003. The second is the Laos Maternal and Child Health and Nutrition (LMCHN) project, implemented by JSI Research and Training Institute, Inc. under Cooperative Agreement #72043921CA00001. Salaries for TF, NV, KG, and SA were funded by MKA. Consultant salary for ES was funded by MKA. Salaries for EB, PP, and BX were funded by LMCHN. BS contributed to this manuscript independently and received no compensation. The funders had no role in study design, data collection and analysis, decision to publish, or preparation of the manuscript.

## Findings

### Demographic characteristics

Sociodemographic data were only collected for the Oudomxay group and not for the pilot participants, as the pilot phase was intended to test and refine the tool rather than contribute to the main analysis. Among the 27 Oudomxay participants, nearly half identified as Khmu (48.1%), 48.1% were Buddhist, and 33.3% had incomplete secondary education (Table 3). Demographic variation provides important context for understanding caregivers’ perspectives on pediatric health care. Ethnicity and religion are reported to characterize the sociocultural diversity of the sample and to contextualize the adaptation work undertaken during cognitive testing, as both factors have been shown to shape health-seeking behaviors and experiences with health services in Laos.

**Table 3:** Demographic characteristics of study respondents in Oudomxay.

Characteristic	n	%
**Ethnicity**		
Khou	13	48.1
Lao	4	14.8
Lue	6	22.2
Tai	1	3.7
Thaineua	1	3.7
Yang	2	7.4
**Religion**		
Animist	11	40.7
Buddhist	13	48.1
Christian	3	11.1
**Education level**		
Completed primary level	5	18.5
Incomplete secondary level	9	33.3
Completed secondary level	7	25.9
Bachelor’s degree	3	11.1
Vocational Training	3	11.1
**Total**	**27**	**100**

### Question clarity

The “question clarity” typology addresses issues related to unclear wording, ambiguous phrasing, or overly complex language that can confuse respondents, hindering their ability to accurately interpret survey items and provide meaningful responses. This domain ensures that questions are straightforward, precise, and easily understood by the target population. In our analysis, we focused on identifying instances where respondents struggled to grasp the intent behind questions, encountered confusing or misleading terms, or faced difficulties in relating the questions to their lived experiences.

One key finding was that questions requiring subjective judgment were difficult for respondents to interpret consistently, particularly within the Lao cultural context. We revised these items for clarity and cultural relevance. Abstract or open-ended questions, such as those about perceptions of wait time or privacy, were especially challenging, as the underlying concepts did not easily translate or hold the same significance in everyday Lao communication. These items were often ambiguous and misaligned with local norms, underscoring the need for culturally grounded phrasing to elicit meaningful responses.

For instance, the original question about “wait times” relied on subjective perceptions, which varied widely. In Laos, where time is not typically discussed in exact units, respondents struggled to interpret what constituted a “long” or “acceptable” wait. Some answered based on emotions (i.e., how they felt at the time), while others referenced the urgency of their child’s condition. To address this, we revised the questions to ask respondents to estimate their wait in minutes, providing a concrete reference point that made subsequent questions about wait time more interpretable (Table 4). Similarly, questions about privacy were problematic due to their subjective nature. To improve clarity, we added a follow-up question to ask if anyone had entered the consultation room during the visit, which was an observable event that respondents could more easily recall.

**Table 4:** Illustrative example of improving question clarity through cognitive testing.

Original question	Rephrased question
Question 1. How long was the amount of time you had to wait from the time that you arrived at the facility to when you were seen by a health care provider during the visit today for your child? Response options: Very short, somewhat short, somewhat long, very long	Question 1a. How many minutes did you wait from the time that you arrived at the facility to when you were seen by a health care worker during the last visit for your child? Response: [Open-ended] Question 1b. What did you think about the amount of time you waited at the facility? Response options: Very short, somewhat short, somewhat long, very long

These revisions replaced abstract or ambiguous concepts with specific, contextually appropriate questions aligned with local communication norms and daily experience. An exchange from pilot testing at Xaythany District Hospital in Vientiane Province illustrates this:

***Interviewer:** “How long was the amount of time you had to wait from the time that you arrived at the facility to when you were seen by a health care provider during the visit today for your child?”*
***Mother:** “It depends on the actual situation. If there aren’t many people, the examination happens quickly. But if there are many patients, then we have to wait longer—depending on the queue.”*
***Interviewer:** “Apart from your given answer, were there any answers you could give? Could you give an approximate waiting time in minutes?”*
***Mother:** “Yes, about 15 minutes.”*
***Interviewer:** “How would you describe a 15-minute wait? Can you give a response?”*
***Mother:** “Fast.”*

Here, the respondent initially answered in narrative terms, focusing on situational context rather than using the predefined response categories (“very short,” “somewhat short,” “somewhat long,” or “very long”). Only after probing did she quantify the time and give an evaluative judgment. This reflects common communication styles in Lao culture, where people may prefer to offer explanations rather than provide definitive, categorical responses, particularly when questions involve subjective assessment. These patterns highlight the importance of culturally sensitive questionnaire design and interviewer training.

### Translation and linguistic accuracy

The “translation and linguistic accuracy” typology focuses on ensuring that survey items are translated in a way that accurately reflects their intended meaning in the target language, while avoiding distortions caused by incorrect or overly literal translations. This domain examines whether the translation aligns with local linguistic conventions and cultural contexts, ensuring that questions are both understandable and culturally appropriate. In our analysis, we specifically looked for instances where direct translations led to confusion, misinterpretation, or loss of meaning, as well as any terms that may not have equivalent or familiar counterparts in Lao, which could affect respondents’ ability to comprehend or engage with the survey.

One finding in this domain was that certain terms resonated more clearly with respondents due to their common usage and understanding in the Lao language and cultural context. For instance, “health worker” was preferred over terms like “health provider” or “health personnel,” as it is more universal and familiar in everyday language, encompassing doctors, nurses, midwives, and other care staff, regardless of their formal qualifications. In contrast, “health provider” and “health personnel” felt too formal and bureaucratic. This subtle difference in terminology improved comprehension and made respondents more comfortable during interviews.

A similar preference was found for the term “health facility.” Respondents understood it better than more specific terms like “hospital,” “clinic,” or “health center,” which carry distinctions related to service level and size that could lead to confusion. For example, if a question mentioned “hospital,” some respondents might assume it excluded their experiences at a health center. Using “health facility” helped avoid misinterpretation and ensured all relevant care settings were included.

We also discovered a key distinction between the terms “feeling” and “thinking,” which revealed the influence of language and cultural norms on understanding. In Lao culture, emotional restraint is valued, and overt expressions of emotion, especially in formal or public settings, are discouraged. Discussing personal feelings with authority figures like health workers or researchers can be uncomfortable. However, expressing thoughts or opinions is more socially acceptable.

This cultural nuance had significant implications for survey instrument design. Questions about emotions could cause discomfort or lead to inaccurate responses, so we revised them to focus on “thoughts” instead of “feelings.” For example, we changed “How did you feel about the care you received?” to “What did you think about the care you received?” (Table 5). This adjustment not only aligned with local communication styles, but also improved clarity, leading to more accurate and thoughtful feedback from respondents.

**Table 5:** Illustrative example of linguistic adaptation from emotional to cognitive framing.

Original question	Rephrased question
Question 13. Did you **feel** you could discuss your child’s problems with the health workers privately, without others not involved in the care overhearing your conversations? Response options: No, never; yes, but rarely; yes, most of the time; yes, all of the time	13. Did you **think** you could discuss your child’s problems with the health workers privately, without others not involved in the care overhearing your conversations? Response options: No, never; yes, but rarely; yes, most of the time; yes, all of the time

This exchange from a caregiver interview at a district hospital in Vientiane province highlights how emotionally-oriented (“feel”) versus cognitively-oriented (“think”) language can impact response clarity and interpretation in health-related interviews:

***Interviewer:** “Did you feel you could discuss your child’s problems with the health workers privately, without others not involved in the care overhearing your conversations?”*
***Mother:** “The health workers didn’t really have a conversation with me. They only asked how my child was and whether I had given any medicine—just regular questions. It didn’t feel private.”*
***Interviewer:** “Let me rephrase this. Do you think you can talk with the health worker privately?”*
***Mother:** “Yes.”*
***Interviewer:** “From your feeling, would you say ‘No never, yes but rarely, yes most of the time, yes all the time’?”*
***Mother:** “Yes, but rarely.”*

This exchange illustrates a key cultural and linguistic nuance in Laos, showing how the emotional framing of questions can influence the interpretation of a respondent’s experience. The initial use of “feel” led the mother to focus on whether the health worker initiated a private conversation, reflecting a situational view of privacy rather than her own perception of it. However, when the interviewer shifted to “think,” the response became more focused on her actual perception of privacy. This distinction highlights how language shapes both the clarity and depth of responses in health interviews.

In collectivist cultures like Laos, emotional self-assessment is often less explicitly verbalized, especially with authority figures like health care workers [39,40]. Privacy may not be understood in the individualized way the original question assumed. Culturally, direct expressions of discomfort or entitlement are discouraged, making emotionally charged terms like “feel” less effective. By rephrasing the question to use “think,” the interviewer helped the respondent assess the possibility of private communication with the provider. This led to a clearer, more direct response, showing that cognitive framing better matched the respondent’s interpretive style. The final answer, “Yes, but rarely,” emerged only after this shift, demonstrating that cognitive framing improved the clarity of responses.

Another finding was that the concept of “permission” can carry unintended meanings, especially when applied to culturally normative behaviors like breastfeeding. In many parts of the world, public breastfeeding may require social approval or explicit permission. However, in Laos, breastfeeding is widely accepted, seen as a mother’s responsibility, and an expected part of child-rearing [41,42]. It is not considered a discretionary act that requires permission, even in public or semi-public spaces like health facilities. While regional and ethnic differences exist, the dominant view is that breastfeeding is a natural right, not something to be regulated.

In this context, the original survey question, “Were you allowed and encouraged to breastfeed or give food or drink to your child while waiting in the facility?” suggested that permission might be necessary, potentially misaligning with local norms and undermining a mother’s autonomy. To better reflect cultural expectations, the question was revised to: “If you wanted to breastfeed or give food or drink to your child while at the facility, were you told to stop or to go somewhere else?” (Table 6). This new phrasing shifts the focus from permission to whether any barriers or discouragement occurred.

**Table 6:** Illustrative example of cultural adaptation of breastfeeding-related items.

Original question	Rephrased question
Question 3. Were you allowed and encouraged to breastfeed or give food or drink to your child while waiting in the facility? Response options: Yes, no.	Question 3. If you wanted to breastfeed or give food or drink to feed your child while at the facility, were you told not to stop or to go somewhere else? Response options: I was told to stop, I was told to go somewhere else, it wasn’t relevant to my last visit.

This revision aligns with national public health initiatives in Laos, where exclusive breastfeeding rates are still below target levels [43]. By focusing on potential interference rather than permission, the updated question better captures cultural norms, supports public health objectives, and ensures more accurate, contextually relevant data collection.

As a final example, the tool included two questions designed to identify potential verbal or physical mistreatment of children by health care providers, with response options as “*no, never”; “yes, once”; “yes, a few times”; “yes, many times”*:

**Q30:** Did the providers shout at, yell, scold, insult, threaten, or talk rudely to your child?
**Q31:** Did the health care workers hit or physically harm your child?

While no major revisions were made to the wording during cognitive testing, important issues emerged during translation and pre-testing. In English, Q30 lists specific forms of negative verbal behavior, each with distinct meanings. However, in Lao, such distinctions are often not made in everyday speech. A more general phrase like *“speaking badly”* is typically used to describe range of disrespectful speech.

This linguistic simplification affected interpretation. During testing, respondents focused more on the provider’s tone and manner rather than distinguishing between specific behaviors like yelling or insulting. As a result, responses were often based on subjective impressions rather than discrete verbal acts of mistreatment. To improve clarity, it may be necessary to provide culturally relevant examples, such as raising one’s voice, blaming the child, or using disrespectful language, to ensure a clearer and more consistent understanding of the question.

In contrast, the question on physical mistreatment (Q31) translated clearly and was widely understood. Actions like hitting or causing pain were easily recognized and consistently interpreted, indicating stronger alignment between English and Lao conceptualizations of physical harm. These findings highlight the importance of linguistic and cultural nuance in question design. Even when the intent is preserved in translation, subtle differences in interpretation can arise, emphasizing the need for culturally grounded adaptations to ensure accurate and meaningful data.

### Cultural appropriateness and relevance

The “cultural appropriateness and relevance” domain examines how well survey items align with local cultural norms, values, and behaviors. In our analysis, we focused on identifying terms, concepts, and questions that may not resonate with or could be misinterpreted due to cultural differences or cultural sensitivities. We looked for instances where survey items either failed to account for local customs, misrepresented culturally sensitive topics, or assumed universal understandings of concepts that may vary significantly across cultures. This domain ensures that the tool captures experiences and perspectives that are both meaningful and relevant within the local context, ultimately improving the validity and reliability of the data collected.

In the hierarchical and collectivist cultural context of Laos, social interactions are strongly influenced by social status, age, education, and professional roles, with deep-rooted expectations around respect and authority. Unlike the Western concept of “mutual respect,” respect in Lao culture is often automatically granted to figures of authority, such as doctors, based on their status, not necessarily their behavior. This cultural norm, particularly among rural and lower-income patients, can make it uncomfortable or inappropriate for individuals to assess whether they were treated respectfully, as deference is a deeply ingrained value in health care interactions [44,45].

This cultural dynamic underpinned many of our findings across all domains, shaping how respondents interpreted questions about respect, authority, and interpersonal interactions in health care settings. It posed challenges for survey questions framed around abstract or Western concepts like “respect.” Research in Laos suggests that younger women, for example, may face discriminatory or dismissive treatment from health workers, but such experiences are difficult to capture with generalized terms like “respect” [46]. To address this, we revised the survey to focus on concrete, culturally relevant behaviors (Table 7), rephrasing questions about “respect” to ask if patients felt “treated with kindness” or “listened to carefully.” These adjustments grounded questions in tangible experiences, improving cultural relevance and the reliability of the data.

**Table 7:** Illustrative example of adapting abstract concepts to observable behaviors.

Original question	Rephrased question
6. Did the health workers treat your child with **respect**? Response options: No, never; yes, but rarely; yes, most of the time; yes, all of the time	6. Did the health workers treat your child with **kindness**? Response options: No, never; yes, but rarely; yes, most of the time; yes, all of the time

The revision of a survey question on “respect” highlights how culturally rooted concepts can impact question interpretation and response accuracy. During tool development, the research team reviewed key terms for cultural and linguistic appropriateness in Laos. Although “respect” has a direct translation in Lao, it carries hierarchical connotations that conflicted with the intended meaning of mutual regard. In Lao society, respect is generally directed toward authority figures and is not expected to be reciprocated, especially toward children. Asking caregivers if health workers showed “respect” to their children created a cultural mismatch, as respondents might have felt unqualified to judge someone in a higher social position.

To address this, “respect” was replaced with “kindness,” a term that is emotionally accessible, culturally resonant, and more suited to describing children’s experiences. Unlike “respect,” “kindness” does not invoke status or hierarchy, making it easier for caregivers to assess whether their child was treated with warmth and care. This change, validated through pilot testing, demonstrates how small lexical adjustments can improve cultural relevance, clarity, and data validity.

Another finding was the issue with the term “trust.” In Laos, trust is often assumed based on social roles and status, rather than developed through mutual exchange. People are generally expected to trust authority figures, like health workers, by default [29,47]. As a result, asking whether a provider “built trust” with a child during a brief clinical encounter felt culturally incongruent.

To improve comprehension, we replaced “building trust” with more concrete and observable language, asking whether the provider made the child feel “calm and comfortable” (Table 8). This phrasing focused on a visible emotional outcome that caregivers could easily interpret, and recognized that trust, as understood in Western contexts, typically develops over time, not in a single visit. By centering on immediate, tangible behaviors, we aligned the survey with Lao cultural norms and improved response accuracy.

**Table 8:** Illustrative example of revising trust-related items for cultural relevance.

Original question	Rephrased question
Question 17. Did the provider build trust with your child before doing procedures? For example, talk to them in a friendly way and explain to them the procedure or ensure they were calm and at ease first. Response options: No never, yes but rarely, yes most of the time, yes all the time	Question 17. Did the health care workers ensure that your child was calm and comfortable and tell them what they were doing before doing procedures or examinations? Response options: No never, yes but rarely, yes most of the time, yes all the time

The following exchange from the cognitive interviews highlights why the original survey item on provider–child trust needed revision. The question asked: *“Did the provider build trust with your child before doing procedures? For example, talk to them in a friendly way, explain the procedure, or ensure they were calm and at ease first.”* While aimed at assessing efforts to build rapport, field testing revealed that the concept of “building trust” was too abstract and culturally misaligned for many respondents in the Lao context.

***Interviewer:** Did the provider build trust with your child before doing procedures? For example, talk to them in a friendly way and explain to them the procedure or ensure they were calm and at ease first. Do you understand the question?*
***Mother:** Yes. My child was calm. Once seeing the health worker, s/he …*
***Interviewer:** Let me rephrase. Did the health worker make an effort to help your child feel calm, comforted, or secure?*
***Mother:** Yes, they made the child feel secure.*
***Interviewer:** Could you explain more? What did the health worker do or say?*
***Mother:** The health worker made the child feel secure. They paid attention.*

In this exchange, the caregiver focused on the child’s emotional state of feeling calm and secure, rather than on specific actions to “build trust.” This suggested that respondents interpreted the question in terms of outcomes rather than provider behavior. It also highlighted a cultural disconnect: in Laos, trust is often implicit and based on a provider’s authority, rather than something actively established through interaction.

In Lao culture, particularly in hierarchical relationships between health workers and patients, trust is typically assumed due to social roles. The idea that a provider should “build trust” with a child may not resonate, and caregivers might feel unqualified to assess such efforts. To address this, the question was revised to: “Did the health worker help your child feel calm, comforted, or secure?” This change used more concrete, observable language, making the question easier for caregivers to understand and respond to while removing the unfamiliar concept of “trust.” The revision led to clearer, more consistent responses, illustrating how culturally grounded cognitive testing can identify and address subtle misalignments in question design, improving both clarity and validity.

Finally, the data collection tool included several questions to assess caregivers’ perceptions of the care environment:

**Q46:** Was the clinic (room you and your child were in) a comfortable temperature?
**Q47:** Did the room where your child was examined have enough privacy (e.g., curtains, closed door, etc.)?
**Q48:** Did it appear to you that the clinic had enough health workers?

While these questions seem straightforward, findings from the cognitive testing revealed challenges related to cultural relevance and respondent interpretation. Terms like “comfortable temperature,” “enough privacy,” and “enough health workers” are inherently subjective and rely on normative judgments that many respondents, particularly those from rural or lower-education backgrounds, felt unqualified to make.

In the Lao context, where social hierarchy influences perceptions of authority, caregivers, especially those from rural areas, may feel uncomfortable assessing facility conditions. Many saw matters such as staffing levels or room adequacy as issues for professionals, not patients. This reluctance is rooted in cultural norms that discourage critiquing authority or institutions, particularly in health care settings.

Additionally, the abstract language used in these questions made them more difficult to interpret. Terms like “comfortable” or “enough” lack clear reference points, leading to varied responses. To improve clarity and cultural fit, we recommend using more specific language, such as “Was the room too hot or too cold?” instead of “comfortable temperature,” and adding introductory cues like, “From your experience today…” to affirm the legitimacy of the caregiver’s perspective. This example underscores that even well-meaning items may produce unreliable data if respondents are unsure how, or whether, they are supposed to answer.

## Discussion

### Cognitive testing as a foundation for culturally valid experience-of-care measurement

This study demonstrates the critical role of cognitive testing in adapting survey instruments for cross-cultural use, both as a methodological tool and an ethical necessity. By applying a flexible typology framework during analysis, we moved beyond surface-level refinements to engage more deeply with cultural, linguistic, and contextual factors shaping how survey items were interpreted. This approach aligns with Willis and Miller (2011), who emphasize that cognitive testing reveals nuanced issues in cross-cultural research and strengthens instrument validity and reliability [48]. In cross-cultural research, adapting both question phrasing and interviewing techniques is essential to ensure that questions are understood as intended and that responses are meaningful and comparable across participants.

Consistent with prior work, our findings reinforce that cognitive testing is indispensable for identifying cognitive mismatches between survey intent and respondent interpretation that may not be apparent through translation alone [49,50]. Even seemingly straightforward translations masked deeper differences in how concepts were understood, underscoring that cognitive testing serves not only as a technical pre-testing step but also as a means of bridging conceptual frameworks across languages and cultures.

### Grounding abstract experience-of-care constructs in observable behaviors

Across multiple domains, caregivers had difficulty responding to survey items framed around abstract constructs such as “respect,” “trust,” or “comfort.” Similar to findings from Bangladesh and other low-resource settings, respondents required contextual examples and concrete framing to interpret such concepts as intended [32]. Rather than rewriting the tool for each setting, targeted linguistic and conceptual adjustments were sufficient to enhance clarity, cultural relevance, and measurement validity.

The repeated need to rephrase items related to subjective experiences, such as respect, privacy, and wait time, illustrates how norms around communication, power, and emotional expression influence interpretation. Survey design often prioritizes internal consistency and psychometric rigor, but these goals can be undermined when questions are culturally misaligned. As noted by Benítez et al. (2018), cognitive testing is well suited to identifying construct-, item-, and method-level biases that affect response validity [51]. Reframing abstract constructs into observable, behaviorally anchored indicators improved comprehension and response consistency while preserving conceptual intent.

### Language, emotional framing, and response validity in hierarchical settings

Cognitive testing also provided insight into the role of language, tone, and interviewer technique in shaping responses. Bilingual interviewers were able to identify nuanced meanings and propose alternate phrasings that aligned better with local usage, echoing findings that high-quality cognitive interviews depend on culturally attuned facilitators capable of recognizing both linguistic and pragmatic cues [31]. Beyond translation accuracy, interviewers’ ability to navigate conversational norms, such as deference to authority or avoidance of negative statements, proved essential for eliciting authentic responses.

These findings highlight that cultural adaptation requires attention not only to wording but also to how questions are asked and interpreted within specific social contexts. Cognitive testing is increasingly recognized as a validity-enhancing process essential for ensuring comparability across populations [33]. Without such adaptation, survey data risk reflecting researcher assumptions rather than respondent realities [31].

### Authority, deference, and implications for caregiver-reported data

The challenges observed in this study were not random but reflected systematic differences in cultural logic, linguistic conventions, and health care expectations. In the hierarchical social context of Laos, social interactions are strongly shaped by age, socio-economic status, gender, and professional roles, with deep-rooted expectations of respect and deference toward authority figures. Such dynamics can make it uncomfortable or inappropriate for caregivers, particularly those from rural or lower-income backgrounds, to assess or critique provider behavior or facility conditions.

These dynamics have important implications for interpreting caregiver-reported experience-of-care data. Reluctance to evaluate authority figures may contribute to socially desirable responses or limited variability in certain domains. By emphasizing concrete experiences rather than evaluative judgments, the PEC tool mitigates some of these challenges, though social desirability bias may persist. The need for rigorous pretesting and adaptation is further amplified in settings like Laos, where linguistic and cultural diversity may contribute to variation in response patterns across subpopulations [52].

### Implications for adapting experience-of-care measures in low-resource settings

These findings have broader implications for global health measurement. As countries are increasingly required to report on care quality and patient experience, survey tools must reflect the lived realities of respondents. Without contextual alignment, data may obscure rather than reveal service delivery gaps. Our approach demonstrates how survey adaptation can evolve from a procedural task into an iterative process anchored in local knowledge.

The study also highlights the limitations of directly importing conceptual frameworks from high-income contexts into lower-resource settings. Constructs such as mutual respect or emotional evaluation may be valid in their original context but require reformulation to avoid misinterpretation or discomfort. Reframing such constructs into tangible, behaviorally anchored terms can preserve conceptual intent while improving cultural resonance and data quality, consistent with cross-cultural methodological recommendations [53]. Importantly, this approach positions respondents as active interpreters whose perspectives and communication norms shape the meaning of survey questions, reinforcing the ethical imperative of culturally responsive research practice [36].

### Strengths and limitations

A key strength of this study lies in the systematic, theory-informed application of cognitive testing to adapt a survey tool for a culturally distinct setting. Although the response error typology was applied retrospectively, it provided a structured framework for analyzing findings and improved the rigor and transparency of the adaptation process [36]. Attention to cultural nuance supported conceptual localization while preserving the instrument’s underlying framework. Future quantitative studies may wish to examine whether comprehension or response patterns differ systematically across ethnic and religious groups, particularly given the diversity of these characteristics in Oudomxay Province.

Several limitations should be acknowledged. Cognitive testing is qualitative and relies on relatively small sample sizes, which may not capture the full range of variation across subgroups. Interviewer interpretation introduces subjectivity, particularly when response issues span multiple domains. Additionally, adaptations to improve cultural fit may alter how constructs are operationalized, creating trade-offs between cultural appropriateness and cross-context comparability [35]. The study focused on adult caregivers’ interpretations and did not directly capture children’s perspectives, which should be considered in future adaptations.

Cognitive testing improves comprehension and interpretability but does not eliminate all sources of bias. Social desirability bias may emerge during survey implementation, particularly in collectivist and hierarchical contexts such as Laos [54–56]. Respondents may be reluctant to express criticism of health workers or facilities, underscoring the need for culturally attuned approaches to interpreting and mitigating response bias [39]. The purposive selection of study sites in collaboration with PHO and the convenience sample in the pilot phase may have introduced selection bias, as participating facilities may differ from non-participating facilities in ways that could affect the generalizability of findings. Future studies should consider more systematic facility selection approaches where feasible. In addition, the Vientiane pilot phase relied on a small convenience sample of caregivers present during facility visits, which may have introduced selection bias and limited the representativeness of pilot-phase findings. However, this approach was used only for initial tool refinement during interviewer training; participant selection in the main Oudomxay study was conducted through random selection from facility-generated eligibility lists.

## Conclusion

This cognitive testing study represents one phase of a broader program of work to strengthen PEC measurement in LMIC settings. Future research should evaluate the tool’s performance across additional country and cultural contexts, particularly in sub-Saharan Africa and other multilingual settings where norms surrounding communication, authority, and caregiving may differ substantially from those observed in Laos. Such work will be important for refining cross-cultural applicability while preserving the contextual relevance necessary for meaningful experience-of-care measurement.

The study confirms the feasibility and necessity of contextually grounded PEC measurement in low-resource settings and reinforces the importance of iterative, culturally responsive instrument development to ensure meaningful, actionable data for improving child health services.

## Supporting information

S1 File Original and testing versions. (DOCX)

S2 File Final tool. (DOCX)

S1 Checklist Inclusivity in global research. (DOCX)
