# Supplementary material for: Development of a survey tool to measure pediatric experience of care: Cognitive testing and validation in the Laos
Source: PLOS Glob Public Health. 2026 Jun 30;6(6):e0006108. doi: 10.1371/journal.pgph.0006108 (PMC13318006; doi:10.1371/journal.pgph.0006108)
Supplement: S1 File — (DOCX) [file pgph.0006108.s001.docx]

**Scale to Measure Pediatric Experience of Care**

BACKGROUND INFORMATION QUESTIONS:

**Date of interview:**

**Start time of interview:**

**End time of interview:**

**Recording number:**

DEMOGRAPHIC QUESTIONS FOR RESPONDENT TO ANSWER:

**Which province does the respondent live in:**

**Which village does the respondent live in**:

**Age of caregiver**:

**Caregiver occupation**:

**Caregiver level of education (circle)**: None Attended primary, not completed Completed primary Attended secondary, not completed Completed secondary Technical Tertiary

**Ethnicity (circle):**

Lao Phouthay Tai Lue Ngoaun Yang Xaek Thaineua Khmou Pray Xingmoun Phong Thaen Oedou Bid Lamed Samtao Katang Makong Tri Yrou Triang Ta-oy Yae Brao Katu Harak Oy Griang Cheng Sadang Xuay Nhaheun Lavy Pacoh Khmer Toum Guan Moy Kree Brou Akha Pounoy Lahou Syla Hayi Lolo Hor Hmong Ewmien No Response Don’t Know Other

**Caregiver religion:** Buddhist Animist Christian Catholic Muslim Hindu None Other

**Do you speak Lao-Tai**: None A little A lot Fluent

**When answering the next questions, please thinking of the last child you took to the health facility:**

Child’s age (last child you took to the health facility):

Child’s sex:

Does your child have physical or mental disability?

Was the child born at a health facility?

When did you last seek care for the child?

Why did you seek care for the child during the last visit?

What level of health facility did you last seek care for your child (circle one): provincial hospital, district hospital, health facility

| No. | Initial survey version – Version 1 | Version 2 | Version 3 | Version 4 | Version 5 | Q on Final |
| --- | --- | --- | --- | --- | --- | --- |
| 1Q | How long was the amount of time you had to wait from the time that you arrived at the facility to when you were seen by a healthcare provider during the visit today for your child? | a. How long did you wait from the time that you arrived at the facility to when you were seen by a healthcare worker during the last visit for your child?  [Open-ended question]  b. What did you think about the amount of time you waited at the facility? | a. How many minutes did you wait from the time that you arrived at the facility to when you were seen by a healthcare worker during the last visit for your child? [Open-ended question]  b. Did you feel you had to wait too long at the facility during your last visit? [no, yes]  c. Did the waiting area have shelter from sun and rain? [no, yes] |  |  | Q501  Q502  Q503 |
| 1R | Very short, somewhat short, somewhat long, very long |  | REMOVED |  |  |  |
| 2Q | Was there a safe and clean area in the facility for your child?  (If prompt needed: if the child wants to play on the floor, if the facility seemed safe for children) | Was there a safe and clean area to play in the facility for your child?  (If prompt needed: if the child wants to play on the floor, if the facility seemed safe for children) |  | a. Was there a safe and clean area to play in the facility for your child?  (If prompt needed: if the child wants to play on the floor, if the facility seemed safe for children)  ADDED b. Was there a room with toys for your child to play in? | a – REMOVED  b. Was there an area or room with toys for your child to play in? | Q504 |
| 2R | No, yes |  |  |  |  |  |
| 3Q | Were you allowed and encouraged to breastfeed or give food or drink to your child while waiting in the facility? | Were you allowed to breastfeed or give food or drink to your child while waiting in the facility? | If you wanted to breastfeed or give food or drink to your child while at the facility, were you told to stop or go somewhere else? | If you wanted to breastfeed or feed your child while at the facility, were you told not to or to go somewhere else? |  | Q505 |
| 3R | No, yes |  | I could feed my child if I wanted to, I was told to stop, I was told to go somewhere else, it wasn’t relevant to my last visit |  |  |  |
| 4Q | How did you feel about the amount of time the main health care worker spent with you and your child? (i.e., was it rushed or did they take their time with you) |  |  |  | How satisfied did you feel about the amount of time the main health worker spent with you and your child? | Q506 |
| 4R | It was just right, it was too long, it was somewhat short, it was very short |  |  |  | Very dissatisfied, dissatisfied, satisfied, very satisfied |  |
| 5Q | Did your providers introduce themselves to both you and your child (if old enough) at the beginning of the visit? | Did the healthcare workers introduce themselves to both you and your child (if child can communicate) at the beginning of the visit? |  | Did the healthcare workers say hello to both you and your child (if child can communicate) at the beginning of the visit? |  | Q507 |
| 5R | No none of them, Yes a few of them, Yes most of them, Yes all of them | No none of the health workers, Yes a few of the health workers, Yes most of the health workers, Yes all of the health workers |  |  |  |  |
| 6Q | Did your providers treat your child with respect?  (Prompt: First define respect in the local context. Then ask about specifics to children: Did the providers talk to your child kindly, was friendly and patient with the child and tried to gain the child’s confidence?) | Did the healthcare workers treat your child with kindness?  (Prompt: First define respect in the local context. Then ask about specifics to children: Did the providers talk to your child kindly, was friendly and patient with the child and tried to gain the child’s confidence?) | Did the healthcare workers treat your child with kindness? |  |  | Q510 |
| 6R | No never, yes but rarely, yes most of the time, yes all the time |  |  |  |  |  |
| 7Q | Did you ever feel that the providers were dismissive of your or child's concerns? | Did you ever feel that the healthcare workers ignored your or your child's concerns? |  |  | Did you feel that the health workers took your concerns about your child seriously? | Q511 |
| 7R | No never, yes but rarely, yes most of the time, yes the whole time |  |  |  |  |  |
| 8Q | Did the providers listen to your observations and experience and knowledge about your child? | Did the healthcare workers listen to your opinions, experience and knowledge about your child? |  | Did the healthcare workers listen to your opinions about your child? |  | Q512 |
| 8R | No never, yes but rarely, yes most of the time, yes all the time |  | No none of the health workers, Yes a few of the health workers, Yes most of the health workers, Yes all of the health workers |  |  |  |
| 9Q | Were you or another family member able to stay with your child as much as you wanted, including during minor medical procedures? | Were you or another family member able to stay with your child as much as you wanted, including during minor medical procedures? |  |  |  | Q514 |
| 9R | No never, yes but rarely, yes most of the time, yes all the time | No never, yes but rarely, yes most of the time, yes all the time |  |  |  |  |
| 10Q | Did you ever have challenges navigating the services in the health facility (e.g., knowing what to do, where to go, who to ask) but had no help to figure it out? | Did you ever have challenges navigating the services in the health facility (e.g. knowing what to do, where to go, who to ask) but had no help to figure it out? | Were you able to navigate the services in the health facility (e.g. knowing what to do, where to go, who to ask)? |  |  | Q513 |
| 10R | No never, yes but rarely, yes most of the time, yes all the time | No never, yes but rarely, yes most of the time, yes all the time |  |  |  |  |
| 11Q | At any point during the visit, did you ever feel that your child was scared or isolated? | At any point during the last visit, did you ever feel that your child was scared or isolated? | At any point during the last visit, was your child was scared or isolated? | At any point during the last visit, was your child left alone? |  | Q537 |
| 11R | No never, yes but rarely, yes most of the time, yes all the time |  |  |  |  |  |
| 12Q | Did you feel your child's health information was kept confidential and private by providers and staff? | Did you feel your child's health information was kept confidential and private by healthcare workers and staff? |  | REMOVED |  |  |
| 12R | No never, yes but rarely, yes most of the time, yes all the time | No never, yes but rarely, yes most of the time, yes all the time | No never, yes but rarely, yes most of the time, yes all the time, I don’t know | REMOVED |  |  |
| 13Q | Did you feel you could discuss your child’s problems with the health workers privately, without others not involved in the care overhearing your conversations? | Did you feel you could discuss your child’s problems with the health workers privately, without others not involved in the care overhearing your conversations? |  |  | REMOVED |  |
| 13R | No never, yes but rarely, yes most of the time, yes all the time | No never, yes but rarely, yes most of the time, yes all the time |  |  |  |  |
| 14Q | Did the providers communicate in a way that you were able to understand? | Did the healthcare workers communicate in a way that you were able to understand? |  | Did the healthcare workers speak in a way that you were able to understand? |  | Q508 |
| 14R | No never, yes but rarely, yes most of the time, yes all the time |  |  |  |  |  |
| 15Q | Did your child’s providers involve you in decisions about your child's care? | Did the healthcare workers involve you in decisions about your child's care? |  | Did the healthcare workers ask for your decisions about your child's care? |  | Q515 |
| 15R | No never, yes but rarely, yes most of the time, yes all the time |  |  |  |  |  |
| 16Q | Did the health workers respect decisions you took about your child’s care, even if your partner (or male family member) was not present? | Did the health workers respect decisions you took about your child’s care, even if your spouse/the parents was/were not present? |  | REMOVE |  |  |
| 16R | No never, yes but rarely, yes most of the time, yes all the time |  |  | REMOVE |  |  |
| 17Q | Did the provider build trust with your child before doing procedures? (For example, talk to them in a friendly way and explain to them the procedure or ensure they were calm and at ease first) | Did the healthcare workers build trust with your child before doing procedures or examinations? (For example, explain to them the procedure or ensure they were calm and comfortable) | Did the healthcare workers ensure that your child was calm and comfortable and tell them what they were doing before doing procedures or examinations? |  |  | Q516 |
| 17R | No never, yes but rarely, yes most of the time, yes all the time |  |  |  |  |  |
| 18Q | Did providers or other staff ask your permission/consent (and your child’s if old enough) before touching your child? (For example, this might be for procedures or examinations) | Did healthcare workers or other staff ask your permission/consent (and your child’s if s/he can communicate) before touching your child for procedures and examinations? |  | Did healthcare workers or other staff ask your permission/consent (and your child’s if s/he can speak) before touching your child for procedures and examinations? |  | Q517 |
| 18R | No never, yes but rarely, yes most of the time, yes all the time |  |  |  |  |  |
| 19Q | Did your providers explain to you why they were giving your child any medicine or vaccination? | Did the healthcare workers explain to you why they were giving your child any medicine or vaccination? |  |  |  | Q518 |
| 19R | No never, yes but rarely, yes most of the time, yes all the time N/A | No never, yes but rarely, yes most of the time, yes all the time, N/A |  |  |  |  |
| 20Q | Did you feel you could ask your providers any questions you had about your child’s care? | Did you feel you could ask your healthcare workers any questions you had about your child’s care? |  |  |  | Q519 |
| 20R | No never, yes but rarely, yes most of the time, yes all the time |  |  |  |  |  |
| 21Q | Did providers encourage you to ask questions about your child’s care? | Did the healthcare workers help you to ask questions about your child’s care? | REMOVED |  |  |  |
| 21R | No never, yes but rarely, yes most of the time, yes all the time |  | REMOVED |  |  |  |
| 22Q | Did your providers ask if your child’s birth had been registered with the authority (and if you needed it, did they provide information about how or why to register their birth)? | Did the healthcare workers ask if your child’s birth had been registered with the authority (and if you needed it, did they provide information about how or why to register their birth)? | Did the healthcare workers ask if your child has a birth certificate (and if you needed it, did they provide information about how or why to register their birth)? |  |  | Q520 |
| 22R | No, yes |  |  |  |  |  |
| 23Q | Did providers give you information to take home at discharge in the easiest way for you to understand (oral, written, pictures, etc.)? | Did the healthcare workers give you medical information to take home at discharge in the way you understand (oral, written, pictures, etc.)? | Did the healthcare workers give you verbal and written care instructions for your child? |  |  | Q521 |
| 23R | No, yes | No, yes, N/A | Yes, written and verbal; Yes, written only; Yes, verbal only; Neither written or verbal |  |  |  |
| 24Q | Did providers check that you understood information that was given to you about your child’s care? | Did the healthcare workers ask that you understood information that was given to you about your child’s care? |  |  |  | Q522 |
| 24R | No never, yes but rarely, yes most of the time, yes all the time |  |  |  |  |  |
| 25Q | Did your child's health providers ask if you have concerns about your child's learning, development or behavior? | Did the healthcare workers ask if you have concerns about your child's learning, development or behavior? |  |  |  | Q523 |
| 25R | No, yes |  |  |  |  |  |
| 26Q | Did providers talk to you about ways to support your child's development (games, speech, therapy, etc.)? | Did the healthcare workers talk to you about ways to support your child's development (games, speech, therapy, etc.)? |  | Did the healthcare workers advise you about ways to support your child's development (games, speech, therapy, etc.)? |  | Q524 |
| 26R | No, yes |  |  |  |  |  |
| 27Q | During your visit, did providers talk to you about ways to keep your child safe at home (reducing burns, drowning, violence, etc.)? | While you were at the facility, did the healthcare workers talk to you about ways to keep your child safe at home (reducing burns, drowning, violence, etc.)? |  |  |  | Q525 |
| 27R | No, yes | No never, yes but rarely, yes most of the time, yes all the time |  |  |  |  |
| 28Q | Do you feel your questions about your child’s care were answered when you did ask? |  |  |  |  | Q526 |
| 28R | No never, yes but rarely, yes most of the time, yes all the time, I didn’t ask any questions |  |  |  |  |  |
| 29Q | Did you hold back on asking questions about your child’s care for any reason? | Were you hesitant to ask questions about your child’s care for any reason? | REMOVED |  |  |  |
| 29R | No never, yes but rarely, yes most of the time, yes all the time |  |  |  |  |  |
| 30Q | Did you feel your providers avoided, ignored, or otherwise neglected your child? | Did you feel the healthcare workers avoided, ignored, or otherwise neglected your child? |  |  |  | Q527 |
| 30R | No never, yes once, yes a few times, yes many times |  |  |  |  |  |
| 31Q | Did the providers shout at, yell, scold, insult, threaten, or talk rudely to your child? | Did the healthcare workers shout at, yell, scold, insult, threaten, or talk rudely to your child? |  |  |  | Q528 |
| 31R | No never, yes once, yes a few times, yes many times |  |  |  |  |  |
| 32Q | Did the providers ever assault, beat, or physically harm your child? | Did the healthcare workers hit or physically harm your child? |  |  |  | Q529 |
| 32R | No never, yes once, yes a few times, yes many times |  |  |  |  |  |
| 33Q | Did you feel that providers blamed you for your child’s illness/condition? | Did you feel that the healthcare workers blamed you for your child’s illness/condition? | Did the healthcare workers blame you for your child’s illness/condition? |  |  | Q531 |
| 33R | No, yes | No never, yes once, yes a few times, yes many times,  child wasn’t sick (well visit) |  |  |  |  |
| 34Q | Did you feel that providers made you feel bad for not coming to the facility sooner? | Did you feel that the healthcare providers made you feel guilty for not coming to the facility sooner? | Did the healthcare workers make you feel guilty for not coming to the facility sooner? | ADDED- Did the health workers make you feel guilty for coming to the health facility too many times? |  | Q532  Q533 |
| 34R | No, yes | No never, yes once, yes a few times, yes many times |  |  |  |  |
| 35Q | Do you feel like your providers ever held your child or restrained them too forcefully? | REMOVED |  |  |  |  |
| 35R | No never, yes once, yes a few times, yes many times | REMOVED |  |  |  |  |
| 36Q | Did you feel like your providers handled your child roughly, pushed, or shoved them? | Did you feel like the healthcare workers handled your child roughly, held them down too strongly, pushed them, or shoved them? | Did the healthcare workers handle your child roughly, held them down too strongly, pushed them, or shoved them? |  |  | Q530 |
| 36R | No never, yes once, yes a few times, yes many times |  |  |  |  |  |
| 37Q | Did the provider provide comfort when the child expressed fear or pain? | Did the healthcare workers provide comfort when the child expressed fear or pain? |  |  | REMOVED |  |
| 37R | No never, yes once, yes a few times, yes many times | No never, yes once, yes a few times, yes many times, child never expressed fear or pain |  |  |  |  |
| 38Q | Did you feel your providers gave the best care they could for your child? | In your opinion, did you feel the health workers gave the best care they could for your child? | In your opinion, did the healthcare workers give the best care they could for your child? |  |  | Q564 |
| 38R | No never, yes but rarely, yes most of the time, yes all the time |  |  |  | No, yes |  |
| 39Q | Would you say your child was discriminated against because of something about your family? (for example: race, ethnicity, composition, number of children, insurance status, religion, immigration status, level of education, or economic status) | Did you feel that your child was discriminated against because of something about your family? (For example: race, ethnicity, marital status, number of children, insurance status, religion, immigration status, level of education, or economic status) |  |  |  | Q534 |
| 39R | No never, yes but rarely, yes most of the time, yes all the time |  |  |  |  |  |
| 40Q | Would you say your child was discriminated against because of their sex, size, health condition, disability or other physical attribute? | Did you feel that your child was discriminated against because of their sex, size, health condition, disability or other physical or mental attribute? |  |  |  | Q535 |
| 40R | No never, yes but rarely, yes most of the time, yes all the time |  |  |  |  |  |
| 41Q | Would you say that your child was discriminated against because their face or clothing was not clean? | Did you feel that your child was discriminated against because their face or clothing was not clean? |  |  |  | Q536 |
| 41R | No, yes | No never, yes but rarely, yes most of the time, yes all the time |  |  |  |  |
| 42Q | Did someone help you to read or write when required, during your time in the clinic? | If you needed help reading or writing, did someone help you during your time in the health facility? |  | If you needed help reading or writing, did a healthcare worker (or other staff) help you during your time in the health facility? |  | Q509 |
| 42R | No, yes, not needed |  |  |  |  |  |
| 43Q | In general, did you feel physically safe in or around the health facility? |  | In general, did you feel physically safe in or around the health facility? (For example, was there any open construction area, traffic, exposed electrical wires, poor lighting or violence?) |  |  | Q538 |
| 43R | No never, yes but rarely, yes most of the time, yes all the time |  |  |  |  |  |
| 44Q | Did you feel that the health facility was sufficiently clean? | Did you feel that the health facility was clean? | Was the health facility dirty? |  |  |  |
| 44R | No never, yes but rarely, yes most of the time, yes all the time | Very clean, somewhat clean, somewhat dirty, very dirty | Very dirty, somewhat dirty, somewhat clean, very clean |  |  | Q539 |
| 45Q | Did the facility have a place with clean water for you and your child to get washed? |  | During your last visit, did the health facility have a place with clean water for you and your child to get washed? |  |  | Q540 |
| 45R | No, yes |  |  |  |  |  |
| 46Q | Was the clinic (room you and your child were in) a comfortable temperature? | Was the health facility (the room that you and your child were in) a comfortable temperature? | a. Were you comfortable with the room temperature in the exam room [yes, no]  b. Did you find the temperature too hot or too cold? [too hot, too cold] |  |  | Q541  Q542 |
| 46R | No never, yes but rarely, yes most of the time, yes all the time | Very cold, somewhat cold, just right, somewhat hot, very hot |  |  |  |  |
| 47Q | Did the room where your child was examined have enough privacy (e.g. curtains, closed door, etc.)? |  | a. Did the room where your child was examined have enough privacy (e.g. curtains, closed door, etc.)?  b. While you and your child were with the health worker, did anyone else walk into the room? [No, yes] |  | a. Did the room where your child was examined have enough privacy (e.g. curtains, closed door, etc.)?  ADDED b. Were your discussions with the healthcare worker private? [No never, yes but rarely, yes most of the time, yes all the time]  c. While you and your child were with the health worker, did anyone else walk into the room? | Q543  Q544  Q545 |
| 47R | No never, yes but rarely, yes most of the time, yes all the time | No, yes |  |  |  |  |
| 48Q | Did it appear to you that the clinic had enough health workers? | In your opinion, did you think that the health facility had enough health workers? | Did you think that the health facility had enough health workers? |  |  | Q546 |
| 48R | No, yes |  |  |  |  |  |
| 49Q | Did you ever feel worried that the provider did not have enough training or skills to take care of your child (e.g. medical student, provider had to repeat procedures many times, etc.)? | Did you feel worried that the healthcare workers did not have enough training or skills to take care of your child (e.g. medical student, healthcare worker had to repeat procedures many times, etc.)? | Did you feel worried that the healthcare workers did not have enough training or skills to take care of your child (e.g. healthcare worker had to repeat procedures many times, etc.)? |  |  | Q547 |
| 49R | No never, yes but rarely, yes most of the time, yes all the time |  |  |  |  |  |
| 50Q | Did you think that the clinic had the proper equipment and medications for your child? | Did you think that the healthcare facility had the proper equipment and medicines for your child? |  | a. Did you think that the healthcare facility had the enough equipment and medicines for your child?  b. Did you think that the healthcare facility had proper equipment and medicines for your child?  [No, yes, I don’t know] |  | Q549  Q550 |
| 50R | No, yes | No, yes, Don’t know |  |  |  |  |
| 51Q | Was there a service, test, or drug your child did not get because you could not pay for it? | a. Was there a service, laboratory test, or medicine your child did not get because you could not pay for it?  b. If yes, which service, laboratory test or medicine did you not get because you could not pay for it? [Open-ended response] |  |  |  | Q551  Q552 |
| 51R | No, yes | No, yes |  |  |  |  |
| 52Q | Were you asked to pay for something (visit, lab, medications, bribe) that was supposed to be free? | Were you asked to pay for something (consultation, laboratory test, medicines) that was supposed to be free? |  |  |  | Q553 |
| 52R | No, yes |  |  | No, yes, I don’t know which services/medicines are free |  |  |
|  |  | **NEW** a. Did you have to pay a health worker extra money (or “incentives”) to get better service? [No, yes]  b. If yes, how many people did you pay, if you don’t mind me asking? [Open-ended response]  c. If yes, what was the total amount that you paid, if you don’t mind me asking? [Open-ended response] |  | a. Did you pay a health worker extra money, “incentives or give in-kind gifts to get better service?  b. If yes, how many people did you pay, if you don’t mind me asking?  c. If yes, what was the total amount that you spent, if you don’t mind me asking? [Open-ended response]  d. If you went to the health facility after working hours, were there healthcare workers there? [No, Yes, I went during working hours]  e. If you went to the health facility after working hours, did you pay an extra fee to receive services? [No, Yes, I went during working hours] | a. Did you pay a health worker extra money, “incentives or give in-kind gifts to get better service?  b. If yes, how many people did you pay, if you don’t mind me asking?  c. If yes, what was the total amount that you spent, if you don’t mind me asking? | Q554  Q555  Q556 |
| 53Q | If you felt your child was mistreated or did not receive the best care, are there steps you could take to file a complaint or hold the facility accountable? |  |  | a. Do you know how to file a complaint, if you needed to?  b. If yes, which channel would you use to file a complaint? [Open-ended response] |  | Q560  Q561 |
| 53R | No, yes, I don’t know |  |  |  | No, yes |  |
| 54Q | Did you feel that you had to give the same information over and over again, either to the same person or to several different people? | Did you feel that you had to give the same information over and over again, either to the same healthcare workers or to several different healthcare workers? |  |  |  | Q548 |
| 54R | No never, yes but rarely, yes most of the time, yes all the time |  |  |  |  |  |
| 55Q | In general, how satisfied were you with how you were treated during your visit? | In general, how satisfied were you with how you were treated during your last visit? | In general, how satisfied were you with how you and your child were treated during your last visit? (COMBINED WITH NEXT – YOU AND YOUR CHILD) |  |  | Q562 |
| 55R | Very satisfied, satisfied, dissatisfied, or very dissatisfied |  |  |  |  |  |
| 56Q | In general, how satisfied were you with how your child was treated during your visit? | In general, how satisfied were you with how your child was treated during your last visit? | COMBINED ABOVE – YOU AND YOUR CHILD |  |  |  |
| 56R | Very satisfied, satisfied, dissatisfied, or very dissatisfied |  | REMOVED |  |  |  |
| 57Q | If cost was not a factor, would you come back to this facility or go elsewhere? |  | If cost was not an issue, would you come back to this facility or go elsewhere? |  |  | Q563 |
| 57R | No, yes | Come back to same facility, Go elsewhere, Unsure |  |  |  |  |
|  | ADDED – During your last visit, did you go after working hours?  If yes, were there health workers available?  If yes, did you have to pay an extra fee to receive services? |  |  |  |  | Q557  Q558  Q559 |
| Additional questions for pediatric patients who received inpatient services. If not interviewing inpatient pediatric patients, SKIP. **NOT INCLUDED IN COGNITIVE TESTING** | | | | | | |
| 58Q | Did you feel that welcomed by the staff when you were in the unit where your child was being cared for? |  |  |  |  |  |
| 58R | No never, yes but rarely, yes most of the time, yes all the time |  |  |  |  |  |
| 59Q | Did you believe that providers were aware of your child's medical history? |  |  |  |  |  |
| 59R | No never, yes but rarely, yes most of the time, yes all the time |  |  |  |  |  |
| 60Q | Did your child’s health care providers explain the plan of care throughout the visit? |  |  |  |  |  |
| 60R | No never, yes but rarely, yes most of the time, yes all the time |  |  |  |  |  |
| 61Q | Did you feel pressured into a decision about your child’s care by providers? |  |  |  |  |  |
| 61R | No never, yes but rarely, yes most of the time, yes all the time |  |  |  |  |  |
| 62Q | Did the facility have a policy of open access visiting hours for parents? (for example, you could visit as much as you wanted; there were no limited visiting hours) |  |  |  |  |  |
| 62R | No, yes |  |  |  |  |  |
| 63Q | Could another family member visit your child as much as you wanted? |  |  |  |  |  |
| 63R | No never, yes but rarely, yes most of the time, yes all the time |  |  |  |  |  |
| 64Q | If you could not stay while your child was inpatient, could you call the facility or ward to get information about your child? |  |  |  |  |  |
| 64R | No never, yes but rarely, yes most of the time, yes all the time, N/A |  |  |  |  |  |
| 65Q | Do you believe your child’s care was coordinated between different providers or different wards? |  |  |  |  |  |
| 65R | No never, yes but rarely, yes most of the time, yes all the time, N/A |  |  |  |  |  |
| 66Q | Before giving your child a new medicine or treatment, how often did the staff explain any possible problem or complication that might occur with the medication or treatment? |  |  |  |  |  |
| 66R | No never, yes but rarely, yes most of the time, yes all the time |  |  |  |  |  |
| 67Q | Did you believe l that providers responded promptly to changes in your child's condition? |  |  |  |  |  |
| 67R | No never, yes but rarely, yes most of the time, yes all the time, N/A |  |  |  |  |  |
| 68Q | Did your providers tell you about changes in your child's condition or treatment as soon as it was possible? |  |  |  |  |  |
| 68R | No never, yes but rarely, yes most of the time, yes all the time, N/A |  |  |  |  |  |
| 69Q | Did your providers ask about your preferences about communicating with your child about their health condition (if they are old enough to understand)? |  |  |  |  |  |
| 69R | No never, yes but rarely, yes most of the time, yes all the time |  |  |  |  |  |
| 70Q | Did your providers ask about your emotional well-being (e.g. ask how you were feeling)? |  |  |  |  |  |
| 70R | No never, yes but rarely, yes most of the time, yes all the time |  |  |  |  |  |
| 71Q | Did the facility offer or allow religious or spiritual support if desired? |  |  |  |  |  |
| 71R | No, yes |  |  |  |  |  |
| 72Q | Was the environment quiet and calm enough to allow your child to sleep ? |  |  |  |  |  |
| 72R | No never, yes but rarely, yes most of the time, yes all the time |  |  |  |  |  |
| 73Q | Did you feel that your child was in a safe environment while in the inpatient ward or intensive care unit? |  |  |  |  |  |
| 73R | No never, yes but rarely, yes most of the time, yes all the time |  |  |  |  |  |
| 74Q | Was there a place where you (or another parent/family member) could stay overnight with the child? |  |  |  |  |  |
| 74R | No, yes |  |  |  |  |  |
| 75Q | Did you feel that you were discharged early because of lack of space or overcrowding? |  |  |  |  |  |
| 75R | No, yes |  |  |  |  |  |
| 76Q | Did you ever feel that you or your child could not voluntarily leave the clinic because of lack of payment? |  |  |  |  |  |
| 76R | No, yes |  |  |  |  |  |
| 77Q | Do you feel you were given enough information about a follow up or next visit? |  |  |  |  |  |
| 77R | No, yes |  |  |  |  |  |
| **OPEN-ENDED QUESTIONS** | | | | | | |
| 1 | During the entire time in the health facility, was there a time you felt most supported? If so, when was it? |  |  |  |  |  |
| 2 | During the entire time in the health facility, was there a time you felt that your child was the most supported?  If so, when was it? |  |  |  |  |  |
| 3 | During the entire time in the health facility, when was the time you felt the least supported? |  |  |  |  |  |
| 4 | During the entire time in the health facility, when was the time you felt that your child was the least supported? |  |  |  |  |  |
| 5 | ADDED - What should be improved about your time at the health facility in order to make your child’s experience better?  Prompt: what should be improved by the facility to show you and/or your child more respect? |  |  |  |  |  |
| 6 | ADDED - Is there something ask that we should ask women/caregivers about, with regard to the care of their young children in health facilities in Laos? |  |  |  |  |  |
