# Supplementary material for: Development of a survey tool to measure pediatric experience of care: Cognitive testing and validation in the Laos
Source: PLOS Glob Public Health. 2026 Jun 30;6(6):e0006108. doi: 10.1371/journal.pgph.0006108 (PMC13318006; doi:10.1371/journal.pgph.0006108)
Supplement: S2 File — (DOCX) [file pgph.0006108.s002.docx]

**TOOL 5: PEDIATRIC EXPERIENCE OF CARE**

| No. | Question | Response | Skip |
| --- | --- | --- | --- |
| **INTRODUCTION** | | | |
| NOTE: Caregivers (male or female) of a child under five years who had a recent well or sick child visit at the facility within the previous 3 months (for the pediatric care interview). Must be age 18 or above.  READ THE FOLLOWING GREETING:  Good day! My name is _____________________. We are here on behalf of the Laos Maternal Child Health and Nutrition (LMCHN) project, with the endorsement of the Department of Hygiene and Health Promotion (DHHP) at the Ministry of Health (MOH) to conduct an assessment to learn more about the health services in this province.  I would like to ask you some questions about your experiences during your last visit with your child for well child or sick child services at a facility to better understand how pediatric care is provided at that facility. The questions usually take about 40 minutes.  Your name and the date of service will not be provided in any shared data, so your identify and any information about you will remain completely confidential.  Please know that the decision to participate in this interview is completely voluntary and that your decision will not affect the services you receive. If at any point you would prefer to end the interview, please feel free to tell me. There is no penalty for refusing to participate or stopping.  The parties conducting the study will keep any personal identifying information you provide strictly confidential. Once information that identifies you has been removed, the remaining information you provide may be shared publicly or with third parties, without additional informed consent.  Do you have any questions about the assessment? Do I have your agreement to proceed? | | | |
| **100** | Do I have your consent to proceed? | No 0  Yes 1 | If NO, END INTERVIEW |
| **SECTION 2: LOCATION IDENTIFICATION** | | | |
| **201** | Date of visit (DD/MM/YY) |  |  |
| **202** | Enumerator Code/Number | (open; two digits) |  |
| **203** | Province  SELECT ONE. | Phongsaly 1  Oudomxay 2  Sekong 3  Savannakhet 4  Salavan 5 |  |
| **204** | District  SELECT ONE. | **Phongsaly**  Phongsaly 1  Mai 2  Somphan 3  Bounnua 4  Bountay 5  **Oudomxay**  Xay 6  Nga 7  Beng 8  Houn 9  Pakbeng 10  **Sekong**  Lamam 11  Kalum 12  Dakchung 13  Thateng 14  **Savannakhet**  Atsaphone 15  Songkhone 16  Phalanaxay 17  Xaibuli 18  Champhone 19  **Salavan**  Saravane 20  Lakhonepheng 21  Toumlane 22  Khongsedone 23  Lao ngarm 24 |  |
| **205** | Village Name | (open) |  |
| **206** | Urban/Rural | Urban 1  Rural with road 2  Rural without road 3 |  |
| **SECTION 3: CLIENT CHARACTERISTICS** | | | |
| **301** | How old are you (in years)? | (open; two digits) |  |
| **302** | Have you ever attended school? | No 0  Yes 1 | If NO, SKIP to Q304 |
| **303** | What is the highest level of school you attended or completed? | Attended primary/did not complete 1  Completed primary 2  Attended secondary/did not complete 3  Completed secondary 4  Attended tertiary or higher/did not complete 5  Completed tertiary or higher 6  Attended/completed technical 7 |  |
| **304** | Are you currently married or living together with a man as if married? | No (single; no partner) 0  Yes, married/in union and living together with partner 1  Yes, married/in union but not living together with partner 2 |  |
| **305** | What is your current occupation? | None/not working 1  Professional, technical or managerial 2  Clerical/office assistant 3  Sales and service 4  Skilled manual labor 5  Unskilled manual labor 6  Domestic service/house maids 7  Agriculture 8  Don’t Know 98  Other (specify) 99 |  |
| **306** | What is your ethnicity?  SELECT ONE. ASK THEM TO USE THE ONE THEY MOST IDENTIFY WITH. | Lao 1  Phouthay 2  Tai 3  Lue 4  Ngoaun 5  Yang 6  Xaek 7  Thaineua 8  Khmou 9  Pray 10  Xingmoun 11  Phong 12  Thaen 13  Oedou 14  Bid 15  Lamed 16  Samtao 17  Katang 18  Makong 19  Tri 20  Yrou 21  Triang 22  Ta-oy 23  Yae 24  Brao 25  Katu 26  Harak 27  Oy 28  Griang 29  Cheng 30  Sadang 31  Xuay 32  Nhaheun 33  Lavy 34  Pacoh 35  Khmer 36  Toum 37  Guan 38  Moy 39  Kree 40  Brou 41  Akha 42  Pounoy 43  Lahou 44  Syla 45  Hayi 46  Lolo 47  Hor 48  Hmong 49  Ewmien 50  No Response 97  Don’t Know 98  Other 99 |  |
| **307** | What is your religion? | None 0  Buddhist 1  Animist 2  Protestant 3  Catholic 4  Muslim 5  Hindu 6  Other (specify) 99 |  |
| **308** | How well do you speak Lao-Tai? | None 0  Some/A little 1  A lot 2  Fluent 3 |  |
| **SECTION 4: LAST VISIT INFORMATION** | | | |
| For this next set of questions, I would like to learn more about the last child under five you took to a health facility for a well child or sick child visit. | | | |
| **401** | How old is the last child you took to the health facility? Please estimate the age of the child when you took them to the health facility. | <1 year 0  1 year (12 -23 months) 1  2 years (24 – 35 months) 2  3 years (36 – 47 months) 3  4 years (48 – 59 months) 4 |  |
| **402** | What is the sex of last child you took to the health facility? | Female 1  Male 2 |  |
| **403** | What is your relationship to the child? | Mother 1  Father 2  Grandparent 3  Other (specify) 99 |  |
| **404** | Does your child have any physical or mental disability? | No/None 0  Physical disability 1  Mental disability 2  Don’t Know/No Response 98 |  |
| **405** | What level of health facility did you last seek care for your child? | Health Center 1  District Hospital 2 |  |
| **406** | When did you last seek care for this child at the health facility? | (date dial) |  |
| **407** | Why did you seek care for this child during the last visit? | Well-child visit (vaccination, growth monitoring, etc.) 1  Sick child visit (diarrhea, fever, cough, other illness, etc.) 2 |  |
| **408** | Could you please tell us specifically what the visit was for? | (open ended) |  |
| **409** | Was this child born at a health facility? | No 0  Yes 1 |  |
| **SECTION 5: SURVEY QUESTIONS** | | | |
| **Now I would like to talk about your last visit with this child to the health facility and your experiences with the health workers and services at that facility.** | | | |
| **501** | How many minutes did you wait from the time that you arrived at the facility to when you were seen by a healthcare worker during the last visit for your child?  60 minutes = 1 hour  90 minutes = 1.5 hours  120 minutes = 2 hours  150 minutes = 2.5 hours | (open) |  |
| **502** | Did you feel that you had to wait too long at the facility during your last visit? | No 0  Yes 1 |  |
| **503** | Did the waiting area have shelter from sun and rain? | No 0  Yes 1 |  |
| **504** | Was there an area or room with toys for your child to play in? | No 0  Yes 1 |  |
| **505** | If you wanted to breastfeed or feed your child while at the facility, were you told not to or to go somewhere else? | I could feed my child if I wanted to 1  I was told to stop 2  I was told to go somewhere else 3  It was not relevant to my last visit (not applicable) 4 |  |
| **Now let’s talk about your interactions with the health care worker who was in charge of the care of your child at the health facility.** | | | |
| **506** | How satisfied did you feel about the amount of time the main health worker spent with you and your child? (i.e., was it rushed or did they take their time with you) | Very dissatisfied 1  Dissatisfied 2  Satisfied 3  Very satisfied 4 |  |
| **507** | Did the health workers say hello to both you and your child (if child can communicate) at the beginning of the visit? | No, none of the health workers 1  Yes, a few of the health workers 2  Yes, most of the health workers 3  Yes, all of the health workers 4 |  |
| **508** | Did the health workers speak in a way that you were able to understand? | No, never 1  Yes, but rarely 2  Yes, most of the time 3  Yes, all of the time 4 |  |
| **509** | If you needed help reading or writing, did a health worker (or other staff) help you during your time in the health facility? | No 0  Yes 1  Not needed 96 |  |
| **510** | Did the health workers treat your child with kindness? | No, never 1  Yes, but rarely 2  Yes, most of the time 3  Yes, all of the time 4 |  |
| **511** | Did you feel that the health workers took your concerns about your child seriously? | No, never 1  Yes, but rarely 2  Yes, most of the time 3  Yes, all of the time 4 |  |
| **512** | Did the health workers listen to your opinions about your child? | No, none of the health workers 1  Yes, a few of the health workers 2  Yes, most of the health workers 3  Yes, all of the health workers 4 |  |
| **Please think about the time you spent at the health facility. We will ask you about who was with you, and who you would have wanted to be with you. We will also ask you about how you felt and how you think your child felt.** | | | |
| **513** | Were you able to navigate the services in the health facility (e.g., knowing what to do, where to go, who to ask)? | No, never 1  Yes, but rarely 2  Yes, most of the time 3  Yes, all of the time 4 |  |
| **514** | Were you or another family member able to stay with your child as much as you wanted, including during minor medical procedures? | No, never 1  Yes, but rarely 2  Yes, most of the time 3  Yes, all of the time 4 |  |
| **Please think about your child’s medical care and how involved you were or wanted to be.** | | | |
| **515** | Did the health workers ask for your decisions about your child’s care? | No, never 1  Yes, but rarely 2  Yes, most of the time 3  Yes, all of the time 4 |  |
| **516** | Did the health workers ensure that your child was calm and comfortable and tell them what they were doing before doing procedures or examinations? | Not Applicable 0  No, never 1  Yes, but rarely 2  Yes, most of the time 3  Yes, all of the time 4 |  |
| **517** | Did health workers or other staff ask your permission/consent (and your child’s if she or he can speak) before touching your child for procedures and examinations? | Not Applicable 0  No, never 1  Yes, but rarely 2  Yes, most of the time 3  Yes, all of the time 4 |  |
| **518** | Did the health workers explain to you why they were giving your child any medicine or vaccination? | Not Applicable 0  No, never 1  Yes, but rarely 2  Yes, most of the time 3  Yes, all of the time 4 |  |
| **519** | Did you feel you could ask your health workers any questions you had about your child’s care? | No, never 1  Yes, but rarely 2  Yes, most of the time 3  Yes, all of the time 4 |  |
| **520** | Did the health workers ask if your child has a birth certificate (and if you needed it, did they provide information about how or why to register their birth)? | No 0  Yes 1 |  |
| **Please think about the information that you got from the health care workers, either during the explanation of their medical care, or at discharge (before you left the facility).** | | | |
| **521** | Did the health workers give you verbal and written care instructions for your child? | Neither written nor verbal 1  Yes, written only 2  Yes, verbal only 3  Yes, written AND verbal 4 |  |
| **522** | Did the health workers ask that you understood information that was given to you about your child’s care? | No, never 1  Yes, but rarely 2  Yes, most of the time 3  Yes, all of the time 4 |  |
| **523** | Did the health workers ask if you have concerns about your child's learning, development or behavior? | No, never 1  Yes, but rarely 2  Yes, most of the time 3  Yes, all of the time 4 |  |
| **524** | Did the health workers advise you about ways to support your child's development (games, speech, therapy, etc.)? | No, never 1  Yes, but rarely 2  Yes, most of the time 3  Yes, all of the time 4 |  |
| **525** | Did the health workers talk to you about ways to keep your child safe at home (reducing burns, drowning, violence, etc.)? | No, never 1  Yes, but rarely 2  Yes, most of the time 3  Yes, all of the time 4 |  |
| **526** | Do you feel your questions about your child’s care were answered when you did ask? | I did not ask any questions 0  No, never 1  Yes, but rarely 2  Yes, most of the time 3  Yes, all of the time 4 |  |
| **Please think about how you feel that you and your child were treated during your last health care visit.** | | | |
| **527** | Did you feel the health workers avoided, ignored, or otherwise neglected your child? | No, never 1  Yes, once 2  Yes, a few times 3  Yes, many times 4 |  |
| **528** | Did the health workers shout at, yell, scold, insult, threaten, or talk rudely to your child? | No, never 1  Yes, once 2  Yes, a few times 3  Yes, many times 4 |  |
| **529** | Did the health workers hit or physically harm your child? | No, never 1  Yes, once 2  Yes, a few times 3  Yes, many times 4 |  |
| **530** | Did the health workers handle your child roughly, hold them down too strongly, push them, or shove them? | No, never 1  Yes, once 2  Yes, a few times 3  Yes, many times 4 |  |
| **531** | Did the health workers blame you for your child’s illness/condition? | No 0  Yes 1  Child was not sick (well child visit) 96 |  |
| **532** | Did the health workers make you feel guilty for not coming to the facility sooner? | No 0  Yes 1 |  |
| **533** | Did the health workers make you feel guilty for coming to the health facility too many times? | No 0  Yes 1 |  |
| **534** | Did you feel that your child was discriminated against because of something about your family? (For example: race, ethnicity, marital status, number of children, insurance status, religion, immigration status, level of education, or economic status) | No, never 1  Yes, but rarely 2  Yes, most of the time 3  Yes, all of the time 4 |  |
| **535** | Did you feel that your child was discriminated against because of their sex, size, health condition, disability or other physical or mental attribute? | No, never 1  Yes, but rarely 2  Yes, most of the time 3  Yes, all of the time 4 |  |
| **536** | Did you feel that your child was discriminated against because their face or clothing was not clean? | No, never 1  Yes, but rarely 2  Yes, most of the time 3  Yes, all of the time 4 |  |
| **537** | At any point during the last visit, was your child left alone? | No, never 1  Yes, but rarely 2  Yes, most of the time 3  Yes, all of the time 4 |  |
| **Please think about the physical environment of the health facility during your last visit, as well as the policies that the facility has.** | | | |
| **538** | Did you ever feel physically unsafe in or around the health facility? (For example, was there any open construction area, traffic, exposed electrical wires, poor lighting or violence?) | Very unsafe 1  Somewhat unsafe 2  Somewhat safe 3  Very safe 4 |  |
| **539** | Was the facility dirty? | Very dirty 1  Somewhat dirty 2  Somewhat clean 3  Very clean 4 |  |
| **540** | Did the health facility have a place with clean water for you and your child to get washed? | No 0  Yes 1 |  |
| **541** | Were you comfortable with the room temperature in the exam room? | No 0  Yes 1 | If YES, SKIP to Q543 |
| **542** | Did you find the temperature too hot or too cold? | Too hot 1  Too cold 2 |  |
| **543** | Did the room where your child was examined have enough privacy (e.g., curtains, closed door, etc.)? | No 0  Yes 1 |  |
| **544** | Were your discussions with the health worker private (e.g., no one else could hear your conversation)? | No, never 1  Yes, but rarely 2  Yes, most of the time 3  Yes, all of the time 4 |  |
| **545** | While you and your child were with the health worker, did anyone else walk into the room? | No 0  Yes 1 |  |
| **Please think about the quality of health care that you think your child received at the health facility during your last visit.** | | | |
| **546** | Did you think that the health facility had enough health workers? | No 0  Yes 1 |  |
| **547** | Did you feel worried that the health workers did not have enough training or skills to take care of your child (e.g., health worker had to repeat procedures many times, etc.)? | No 0  Yes 1 |  |
| **548** | Did you feel that you had to give the same information over and over again, either to the same health worker or to several different health workers? | No, never 1  Yes, but rarely 2  Yes, most of the time 3  Yes, all of the time 4 |  |
| **549** | Did you think that the health facility had the enough equipment and medicines for your child? | No 0  Yes 1  Don’t Know 98 |  |
| **550** | Did you think that the health facility had proper equipment and medicines for your child? For example, small needles, medicine in liquid form (not pills), flat scales for babies, etc. | No 0  Yes 1  Don’t Know 98 |  |
| **551** | Was there a service, laboratory test, or medicine your child did not get because you could not pay for it? | No 0  Yes 1 | If NO, SKIP to Q553 |
| **552** | If yes, which service, laboratory test or medicine did you not get because you could not pay for it? | Service 1  Laboratory test 2  Medicine 3  Other 99 |  |
| **553** | Were you asked to pay for something (consultation, laboratory test, medicines) that was supposed to be free? | No 0  Yes 1  I don’t know which services/medicines are free 98 |  |
| **554** | Did you pay a health worker extra money, incentives, or give in-kind gifts to get better service? | No 0  Yes 1 | If NO, SKIP to Q557 |
| **555** | If yes, how many people did you pay, if you don’t mind me asking? | (open; 1-20) |  |
| **556** | If yes, what was the total amount that you spent, if you don’t mind me asking? (up to 9 digits; no commas; in KIP) | (open) |  |
| **557** | During your last visit, did you go after working hours? | No 0  Yes 1 | If NO, SKIP to Q560 |
| **558** | If yes, were there health workers available? | No 0  Yes 1 | If NO, SKIP to Q560 |
| **559** | If yes, did you have to pay an extra fee to receive services? | No 0  Yes 1 |  |
| **560** | Do you know how to file a complaint, if you needed to? | No 0  Yes 1 | If NO, SKIP to Q562 |
| **561** | If yes, how (which channel) would you file a complaint? | (text) |  |
| **Please think about your overall experience seeking health care for your child during the last visit to the facility.** | | | |
| **562** | In general, how satisfied were you with how you and your child were treated during your last visit? | Very dissatisfied 1  Dissatisfied 2  Satisfied 3  Very satisfied 4 |  |
| **563** | If cost was not an issue, would you come back to this facility or go elsewhere? | Come back to same facility 1  Go elsewhere 2  Unsure 3 |  |
| **564** | In your opinion, did the health workers give the best care they could for your child? | No 0  Yes 1 |  |
| **END OF INTERVIEW. THANK THE RESPONDENT FOR THEIR TIME.** | | | |
