# Supplementary material for: Development of a survey tool to measure pediatric experience of care: Cognitive testing and validation in the Laos
Source: PLOS Glob Public Health. 2026 Jun 30;6(6):e0006108. doi: 10.1371/journal.pgph.0006108 (PMC13318006; doi:10.1371/journal.pgph.0006108)
Supplement: S1 Checklist — (DOCX) [file pgph.0006108.s003.docx]

Inclusivity in global research

PLOS’ policy on inclusivity in global research aims to improve transparency in the reporting of research performed outside of researchers’ own country or community and ensures that PLOS publications reporting global research adhere to high standards for research ethics and authorship. Authors of relevant research articles may be asked to complete the questionnaire below, which outlines ethical, cultural, and scientific considerations specific to inclusivity in global research. This questionnaire may be requested when researchers have travelled to a different country to conduct research, if research uses samples collected in another country, research with Indigenous populations or their lands, or if research is on cultural artefacts. Researchers travelling to another country solely to use laboratory equipment will not normally be required to complete the questionnaire. However, the questionnaire can be requested at the journal’s discretion for any submission – if you have been requested to complete this questionnaire by the PLOS journal you submitted to, please do so.

Please complete the questionnaire below and include this as a Supporting Information file with your manuscript. Note that if your paper is accepted for publication, this checklist will be published with your article in the supporting information files. Please ensure that you reference the checklist in the main body of your manuscript. We suggest adding a subsection ‘Inclusivity in global research’ to your Methods section and adding the following sentence: “Additional information regarding the ethical, cultural, and scientific considerations specific to inclusivity in global research is included in the Supporting Information (SX Checklist)”

The questions have been designed to be applicable to a wide range of study types, and there are subsections for both human subjects research and non-human subjects research. If any of the questions are not relevant to your research please mark them as “N/A” as appropriate.

**Ethical considerations, permits and authorship**

*This section is applicable to all research types.*

Provide details as to who granted permissions and/or consent for the study to take place in the Methods section of your manuscript. This should include the names of **all** ethics boards, governmental organizations, community leaders or other bodies that provided approval for the study. If individuals provided approval refer to these people by their role or title but do not list their name(s).

Reported on page number: **page 15 (clean manuscript)**

**The following information was included in the “Methods: Ethical considerations” section of the manuscript. It was been revised )below) to ensure it is comprehensive and includes additional elements from this inclusivity form.**

***The study was reviewed by the Lao National Ethics Committee for Health Research and determined exempt from full ethical review (Approval #19, 2022). Additional authorization was obtained from the Ministry of Health's Department of Health and Hygiene Promotion, the Department of Healthcare and Rehabilitation, and the Vientiane and Oudomxay Provincial and District Health Offices. All research team members were trained in ethical practices and informed consent, following international standards set by the 1964 Declaration of Helsinki and its later amendments.***

***Participants were caregivers of children under five years of age recruited through health facilities in Vientiane and Oudomxay provinces. No minors were recruited or interviewed. Verbal informed consent, including permission to audio-record, was obtained from each participant in the Lao language before participation began. The consent process, covering the study's purpose, procedures, risks, and voluntary nature, was read aloud to respondents, with consent documented in the survey tool and the interviewer serving as witness.***

If there were any deviations from the study protocol after approval was obtained please provide details of these changes in the Methods section of your manuscript.

**There were no deviations from the approved study protocol.**

**Note that we included this specific clause in the revised “Ethical considerations” sub-section**

Reported on page number: page 15 (clean manuscript)

Did this study involve local collaborators that are residents of the country where the research was conducted or members of the community studied? If you do not have any authors from said communities, please provide an explanation for this below.

**Yes. Three co-authors on this manuscript are Lao nationals and residents of Laos. Two of these co-authors served as co-investigators on the study and were involved in research design, data collection, and fieldwork. The third local co-author contributed to the interpretation of findings and critical review of the manuscript for accuracy, authenticity, and cultural appropriateness. This information is available on page 14 of the clean manuscript.**

Everyone listed as an author should meet PLOS’ criteria for authorship and all individuals who meet these criteria should be included in the author byline, rather than the acknowledgements. For further information please see the journal’s Authorship Policy.

**All authors listed on this manuscript meet PLOS criteria for authorship.**

**Human subjects research (e.g. health research, medical research, cross-cultural psychology)**

Did you obtain written informed consent from a representative of the local community or region before the research took place? How did you establish who speaks for the community? Details of written informed consent obtained from study participants should be reported separately in the Methods section of your manuscript.

**Formal authorization to conduct research was obtained from the Ministry of Health's Department of Health and Hygiene Promotion and the Vientiane and Oudomxay Provincial and District Health Offices, whose approval established permission to engage participants within their jurisdictions. The study was reviewed by the Lao National Ethics Committee for Health Research and determined exempt from full ethical review (Approval #19, 2022) (page 15). Individual verbal informed consent was obtained from all participants in the Lao language prior to their participation, as detailed in the Methods section (page 15). A single community representative was not designated, as authorization was established through the appropriate governmental and health authorities who oversee the health facilities through which participants were recruited.**

How did members of the local community provide input on the aims of the research investigation, its methodology, and its anticipated outcome(s)?

**While community members were not involved in the initial design of the research, local input was incorporated throughout the research process in several ways. Lao national co-investigators and local staff contributed to the design and refinement of the study methodology and data collection instruments. Informal consultations with representatives of the Ministry of Health informed the study's aims and ensured alignment with national health priorities. Cognitive testing was conducted with caregivers of children under five to assess their understanding of the survey questions and response categories, and findings from this testing informed revisions to the tool prior to its application in a health facility assessment.**

When engaging with the local community, how did you ensure that the informed consent documents and other materials could be understood by local stakeholders?

**All study materials, including the informed consent process and survey instrument, were developed in Lao and reviewed by local Lao speakers for accuracy and clarity. The full study protocol was also translated into Lao for submission to and review by the Lao National Ethics Committee for Health Research (NECHR) prior to approval. Consent was administered verbally and read aloud to each participant by trained local research staff, ensuring comprehension regardless of literacy level. The survey tool was further refined based on findings from the cognitive testing, which assessed caregivers' understanding of the questions and response categories.**

Will the findings of the research be made available in an understandable format to stakeholders in the community where the study was conducted (e.g. via a presentation, summary report, copies of publications, etc.)? Please provide details of how this will be achieved.

**Following data collection, findings from the cognitive testing were debriefed with local officials. The findings from this cognitive testing study informed the design of a subsequent, larger health facility assessment conducted in Laos. While that assessment was a separate study, the cognitive testing described in this manuscript was a critical precursor that ensured the survey tool was appropriate and comprehensible for the target population. Findings from the larger health facility assessment were disseminated to local, provincial, and central level officials through presentations and a formal report. The publication of this manuscript further contributes to making the cognitive testing findings accessible to a broader audience of researchers and practitioners working in similar contexts.**

**Non-human subjects research using specimens/ animals collected as part of the study, or those housed in archival collections. Examples include archaeology, paleontology, botany and zoology.**

Did the permission you obtained from a local authority to perform the study include an agreement on access to outputs and benefit sharing? This may include procedures to enable fair distribution of the benefits and resources arising from the research performed. Please include any details of Prior Informed Consent and Benefit Sharing Agreements obtained. These may be required by field-specific regulations, for example the Convention on Biological Diversity (CBD) and the associated Nagoya Protocol.

**N/A; we did not conduct non-human subjects research.**

If the material used in your study was imported, please A) provide the year it was imported and B) indicate whether permits were obtained to import/export the materials used, C) provide details of any permits obtained. If this information is not available, please indicate this.

**N/A; we did not conduct non-human subjects research.**

If you used archival specimens, please state how the material used in your study was acquired by the institute it is held in and provide details of any permits obtained for the original excavations/ sample collection. If this information is not available, please indicate this.

**N/A; we did not conduct non-human subjects research.**

How was the potential cultural significance of the materials collected in your study to local communities considered in your research design? Were Indigenous peoples and/or local researchers and institutions involved with archaeological excavations / collection of specimens? If so, please provide a description of their involvement.

**N/A; we did not conduct non-human subjects research.**

If your manuscript includes photographs of human remains please indicate whether authors obtained permission from descendants or affiliated cultural communities to do so.

**N/A; we did not conduct non-human subjects research.**
